# H-EM: An algorithm for simultaneous cell diameter and intensity quantification in low-resolution imaging cytometry

**DOI:** 10.1371/journal.pone.0222265

**Published:** 2019-09-12

**Authors:** Esteban Pardo, Germán González, Jason M. Tucker-Schwartz, Shivang R. Dave, Norberto Malpica

**Affiliations:** 1 Medical Image Analysis and Biometry Lab, Universidad Rey Juan Carlos, Móstoles, Madrid, Spain; 2 Madrid-MIT M+Visión Consortium, Massachusetts Institute of Technology, Cambridge, MA, United States of America; The Ohio State University, UNITED STATES

## Abstract

Fluorescent cytometry refers to the quantification of cell physical properties and surface biomarkers using fluorescently-tagged antibodies. The generally preferred techniques to perform such measurements are flow cytometry, which performs rapid single cell analysis by flowing cells one-by-one through a channel, and microscopy, which eliminates the complexity of the flow channel, offering multi-cell analysis at a lesser throughput. Low-magnification image-based cytometers, also called “cell astronomy” systems, hold promise of simultaneously achieving both instrumental simplicity and high throughput. In this magnification regime, a single cell is mapped to a handful of pixels in the image. While very attractive, this idea has, so far, not been proven to yield quantitative results of cell-labeling, mainly due to the poor signal-to-noise ratio present in those images and to partial volume effects. In this work we present a cell astronomy system that, when coupled with custom-developed algorithms, is able to quantify cell intensities and diameters reliably. We showcase the system using calibrated MESF beads and fluorescently stained leukocytes, achieving good population identification in both cases. The main contribution of the proposed system is in the development of a novel algorithm, H-EM, that enables inter-cluster separation at a very low magnification regime (2x). Such algorithm provides more accurate brightness estimates than DAOSTORM when compared to manual analysis, while fitting cell location, brightness, diameter, and background level concurrently. The algorithm first performs Fisher discriminant analysis to detect bright spots. From each spot an expectation-maximization algorithm is initialized over a heterogeneous mixture model (H-EM), this algorithm recovers both the cell fluorescence and diameter with sub-pixel accuracy while discriminating the background noise. Finally, a recursive splitting procedure is applied to discern individual cells in cell clusters.

## Introduction

The focus of cytometry is to classify cell types by analyzing physical and molecular biomarkers. Flow cytometers, the preferred instrument for cytometry, utilize photometry techniques to measure cell biomarkers, such as cell diameter and antigen expression, through scattering and fluorescence interactions with laser beams [1]. Cell diameter is usually estimated by measuring the amount of light scattered in the direction of the light beam [1], whereas the expression of specific antigens is estimated by measuring the light emitted by fluorophores bound to such antigens [1]. Even with the development of personal cytometers, cytometry faces challenges including instrumental cost, complexity, and inability to distinguish cell clusters.

An alternative to flow cytometry is fluorescent microscopy and slide scanners to estimate the same physical and biological parameters. Microscopy has made remarkable advances in quantitative molecular detection at typical magnifications (>10x) and has even moved past the diffraction limit for single molecule detection [2]. However, in these magnification regimes, a limited number of cells can be simultaneously imaged per field of view, restricting the throughput of the system.

For many clinically relevant cytometric assays, such as CD3/CD4 counts for monitoring HIV progression, the required clinically actionable information is limited to cell diameter and molecular biomarker expression. For these situations high magnification microscopy, which provides a window into cell morphology, is not required. Shapiro et al. proposed replacing flow cytometry with celular astronomy (imaging cytometry conducted at low magnification, around 4x), due to the inherent lower instrumental complexity [3, 4]. Despite reducing hardware complexity, image quantification at low magnifications poses image analysis problems not typical for higher magnification microscopy such as a) the finite discretization of cells into a small number of pixels, which leads to significant partial volume effects; b) the presence of unbound fluorophores due to sample preparation protocols that do not include wash steps, decreasing the contrast between the signal and the background; c) low fluorescence intensities, which, in combination with the image noise and the background fluorescence, creates a low SNR scenario; and d) cells may be clustered together, complicating the identification and quantification of individual cells. Given the promise of cell astronomy for improving access to clinical cytometry in low-resource settings, these image analysis challenges motivate the development of an automated computer vision algorithm to reliably analyze such low-magnification images. Powered by such algorithms, cell astronomy may therefore by expanded to more advanced cytometric applications.

**Fig 1:**
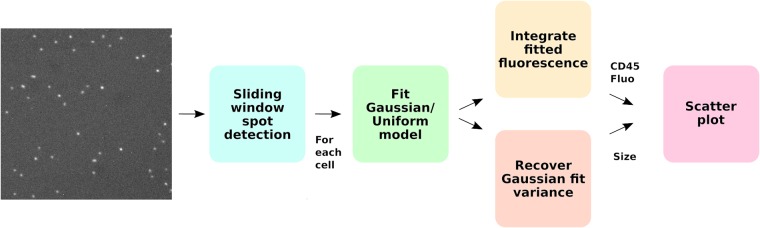
Graphical overview of the proposed approach. The method first detects bright spots using an LDA classifier, then, a model comprised of a gaussian and an uniform distribution is fitted to an image patch around each cell. Finally, the relative contribution of the gaussian and uniform distribution is used to compute the fluorescence intensity, while the variance of the gaussian distribution is used as a diameter estimate. These two features enable the creation of scatter plots similar to the ones resulting from flow cytometry analysis.

An automated algorithm for cell astronomy needs to solve the following tasks: a) locating cells in the image, a task often referred to as “spot detection”; b) estimating the brightness of the cells, a task referred to as photometry; c) estimating the diameter of the cell; and d) finding spots that correspond to multiple cells in close physical proximity to each other, and if such is the case, splitting them into individual events (often referred to as “split and merge”). To solve these challenges, an image processing pipeline was developed which is illustrated in Fig 1. For the initial task of spot detection, we employ standard algorithms from this widely studied topic in automated fluorescence microscopy quantification [5–12]. This task involves the identification of each spot in the image, usually by returning a coordinate related to the spot location or a bounding box. A review by I. Smal et al [5] has shown that supervised machine learning based spot detection methods usually outperform unsupervised ones. Motivated by these results, we have incorporated a Fisher discriminant analysis-based spot detector to the proposed pipeline. If only the presence of cells is needed for the cytometry assay, spot-detection techniques might suffice, as is the case in the diagnosis of infectious diseases by means of CD4+ T cell counting [13]. However, the diagnosis of other conditions such as leukemia requires the analysis of multiple cell biomarkers to adequately distinguish leukocytes cell types by means of a combination of their physical and molecular biomarkers [14], which cannot be obtained using spot detection techniques alone.

The second task is to perform photometry of the under resolved spots detected in the first step of the algorithmic pipeline. We build upon known photometry techniques for the analysis of astronomy images to perform fluorescence and diameter analysis of every bright spot in our images. Typical photometry algorithms assume that every bright spot is the result of observing an unresolved object through a point spread function (PSF) and use an estimate of the point spread function to fit the intensity of every detected bright spot. These kind of photometry algorithms help estimate the luminosity, distance, or chemical composition of astronomical objects [15]. However, they do not typically estimate the diameter of the object since the underlying assumption is that objects are completely unresolved. The most notable example of this family of algorithms is DAOPHOT [16]. This algorithm performs photometry by first calculating the PSF empirically and then fits the location and intensity of every star. For the estimation of the PSF, DAOPHOT considers the width of the gaussian function as a parameter to estimate. Once the PSF is estimated, DAOPHOT only fits the location and brightness of each star, leaving out information that would be useful for cellular analysis such as the spot (cell) diameter.

In microscopy, PSF fitting has been widely used in super-resolution microscopy to locate molecules with super resolution accuracy [2, 17–20]. Following a similar approach to photometry techniques for astronomy images, super-resolution location algorithms perform a spot detection step and then fit an approximation of the PSF to each spot, based on the underlying assumption that each observed spot is the result of applying the point spread function to an unresolved molecule. The super-resolution location accuracy is achieved since the fitted PSF has higher resolution than the image and the fitting is performed in the PSF space [21]. Contrary to PSF fitting in astronomy, the main goal in super-resolution microscopy is to have a highly accurate estimate of the location of each molecule; because of such narrow goals, some authors go as far as to state that fitting a gaussian approximation of the PSF can provide information beyond the desired location of the particle center, such as the amplitude and width of each spot [19].

In recent years, approaches related to the one described have been reported in the field of super-resolution and single-molecule microscopy [22–24]. While photometry in both astronomy and super-resolution microscopy are well studied, their basic premise, that the object being measured is unresolved (i.e., its diameter is mapped to less than a pixel), does not hold for accurate quantification of cells in cellular astronomy—therefore, the above mentioned algorithms are not readily applicable to the problem addressed in this paper. Under cellular astronomy regimes, the cell is imaged to a few number of pixels, which enables the estimation of cell diameter, an important cell biomarker.

In the field of imaging cytometry, several methods for detecting cell shape and fluorescence have been recently described [25–27]; however, these approaches require higher magnification (e.g. 10x). The diameter of cells on the imaging sensor in this technique are much larger than in the cell astronomy regime, and thus the required fitting algorithms are relatively simple.

The goal of the proposed algorithmic pipeline is to mimic the cell biomarker analysis conducted by a flow cytometer, with a higher throughput than that allowed by high magnification cell cytometers. Our method consists of two distinct processes: first, events (bright spots) in the image are detected using Linear Discriminant Analysis and classic sliding window techniques [5], and second, detected events are analyzed using the H-EM algorithm to enable quantification of fluorescence intensity and estimation of the physical diameter. The H-EM algorithm performs expectation maximizations (EM) over a heterogeneous mixture model and has two main novel contributions. First, it uses a uniform distribution to model image noise locally while simultaneously fitting the bright spot with a gaussian distribution. Second, the EM model splits each detection event recursively according to information in the image in order to separate cell clusters into individual cells. This recursive process stops when the Bayesian information criterion does not decrease below a given threshold that is estimated using cross validation.

Finally, the simultaneous estimation of fluorescence intensity and physical diameter enables the differentiation of monocytes, lymphocytes, and granulocytes in images of CD45 labeled white blood cells. Such differentiation is not possible using only fluorescence information, since the marker is non-specific for these cell types.

## Materials and methods

### Microbeads

To demonstrate initial proof-of-concept of the pipeline, microbeads were used as cell surrogates, since they are widely used in flow cytometry to perform quality control, optimize flow cytometer parameters, and measure sample concentration. Their popularity is attributed to their uniformity in diameter and fluorescence intensity, which removes biological sources of variability from the sample. In this study, MESF beads (molecular equivalence of soluble fluorochrome, Bangs Laboratories, Indiana, USA) of fluorescent levels 2, 3, and 4, and diameter between 7.1 and 7.9 *μm*, were used to characterize the accuracy of fluorescent intensity and physical diameter estimation results of the proposed approach. A drop of beads at a concentration of 2.0e6 particles per mL was placed in a microscopy slide and sealed with a cover slip and nail polish.

### Cell controls and sample preparation

Immunophenotyping controls, stabilized blood cell samples that are also widely used in flow cytometry due to their characterized levels of cell types, were used to evaluate the algorithm pipeline in a clinically-relevant use case. CD-Chex Plus (Streck, Omaha, NE) samples were lysed to remove red blood cells and FITC-labeled fluorescent anti-CD45 antibodies following standard protocols. Specifically, two preparations of 500 microliters (2.72e4 white blood cells per microliter) of Streck CD-Chex Plus normal lymphocyte suspension was added to 25 microliters (0.3 micrograms) of FITC-labeled anti-CD45 antibody (AbCam Bio, Cambridge, MA) and incubated at room temperature in the dark for 25 minutes. After incubation, the samples were transferred to 5 mL of 1X red blood cell (RBC) lysis buffer (eBioscience Inc., San Diego, CA) and incubated for 12 minutes at room temperature. The lysis reaction was quenched by diluting the sample with 9 mL of flow cytometry buffer (eBioscience Inc., San Diego, CA), after which the cell samples were isolated by centrifugation at 500 x g at room temperature for 5 minutes followed by decanting of the supernatent and resuspension in 9 mL of flow buffer, twice, and finally resuspended in 500 microliters of flow buffer to obtain the original cell concentration. Both samples were combined and filtered to yield 1 mL with an approximate final cell concentration of 5-10e6 cells per mL. Prior to cell sorting, sample was diluted with 0.5 mL of flow buffer. Granulocytes, monocytes, and lymphocytes were sorted to obtain a cell-subtype reference standard at the Koch Institute Flow Cytometry Core at the Massachusetts Institute of Technology on a FACS Aria III (BD Biosciences, San Jose, CA) running BD FACS Diva software. The cell sorts were split into 2 portions. The first was used for imaging and the second was re-sorted to verify purity of the cell population. The cell control had a population of 7.7% monocytes, 36.0% lymphocytes and 51.4% granulocytes. Sorted samples of lymphocytes, monocytes, and granulocytes were concentrated by centrifugation at 500 x g for 5 minutes at room temperature. To prepare samples for imaging, 10 microliters of sample was pipetted onto cleaned microscope slides and covered with a coverslip. Two images for each of the 5 fields of view were imaged with 5 second integration time, observing an average of either 897 granulocytes, 173 monocytes, or 942 lymphocytes per field of view (FOV). To demonstrate the performance of the imaging and analysis pipeline, we used the proposed H-EM algorithm to analyze the proposed dataset, which took an average of 10.12 seconds per FOV, and compared the results to standard photometry algorithms, and flow cytometry.

### Microscope

**Fig 2:**
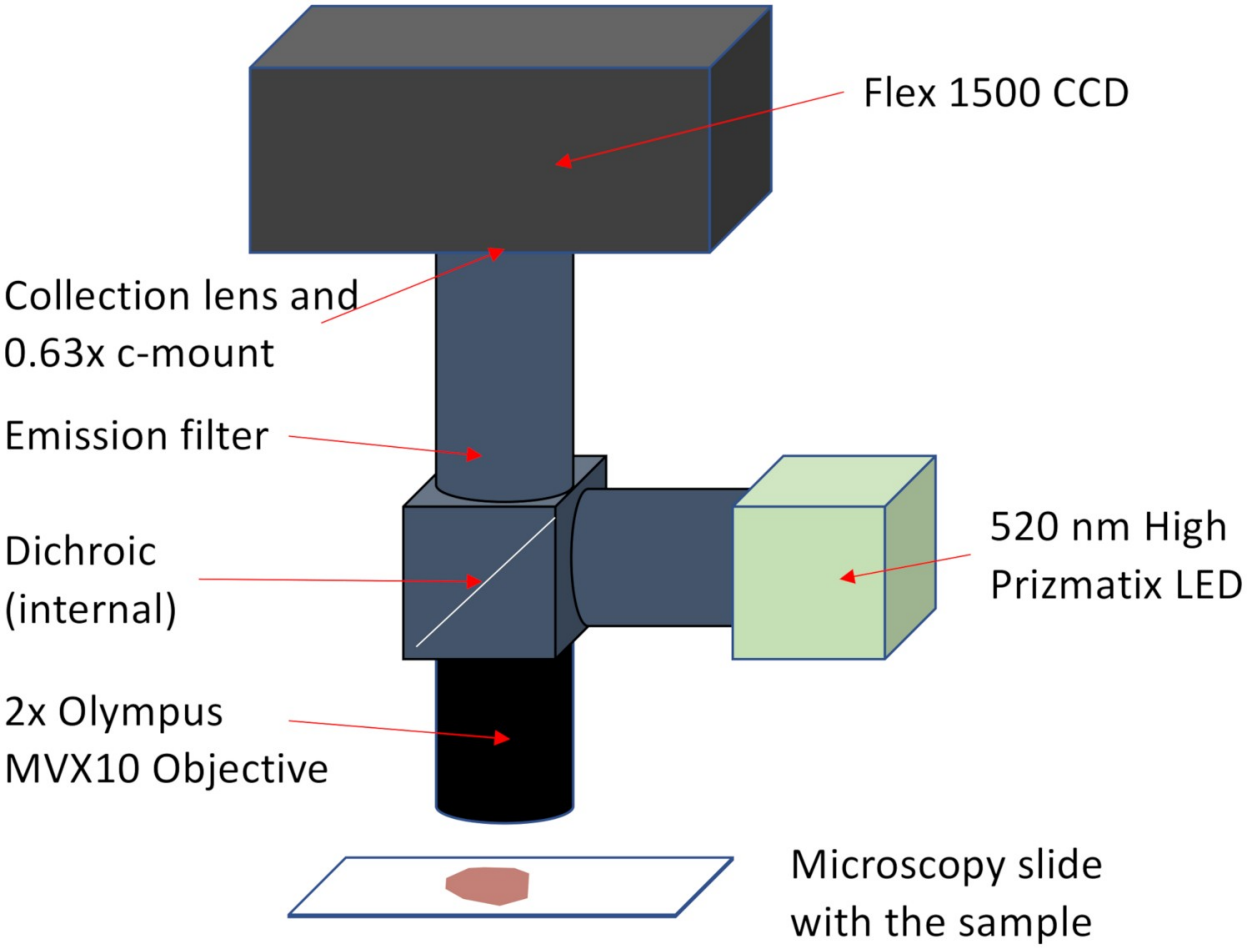
Microscope system. To achieve low magnification and large field of view we used a 2x MVX10 Olympus Objective, a high-power Prizmatix LED and a Flex 1500 Spot Camera. The total maginification of the system is 2.52x, with a resolution of 3*μm* per pixel.

All samples were imaged using a custom-built epi-fluorescence microscope with a a 520 nm high power LED illuminator (UHP-T-520-EP Prizmatix, Holon, Israel). The CCD sensor was a 2048 by 2048 pixel 14 bit CCD camera (Flex 1500, Spot Imaging) with a pixel size of 7.4 *μm*. Each image was acquired using an exposure time of 10 seconds, a gain factor of 1, and a 4x, 0.13 numerical aperture fluorite objective (MVX10, Olympus) coupled to a 0.63x CCD coupler. The resulting total magnification of the system is approximately 2.52x, and the image resolution is approximately 3 *μm* per pixel. A diagram of the system is shown in Fig 2.

### Spot detection

**Fig 3:**
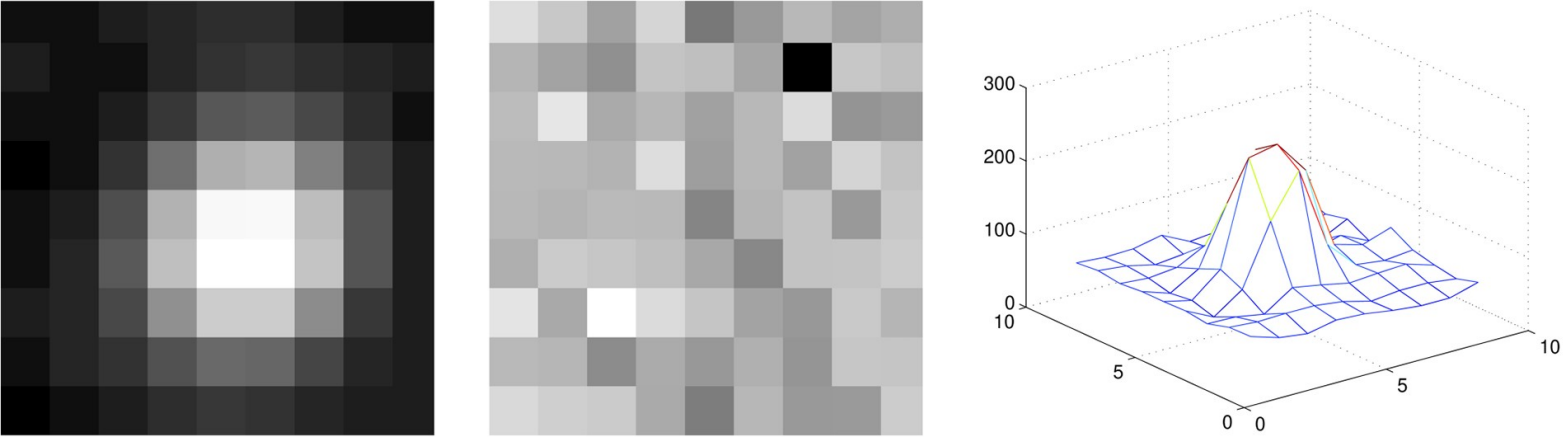
LDA prototypes for cell and background and mesh showing the topology of a cell. The images show the result of averaging patches containing a cell in the center (left), and containing background information (middle). Cells show a gaussian-like distribution while noise follows a uniform distribution (right).

The spot detection algorithm uses a sliding window approach where each window is classified for the presence or absence of a spot. Following the work of [5] the Linear Discriminant Analysis (LDA) algorithm was chosen because it provides a good balance between classification performance, evaluation speed, and conceptual complexity. The classification algorithm is trained using a set of manually labeled spot locations. Image patches around each cell location are extracted and normalized with respect to the intensity levels. The chosen patch size was of 15x15 pixels. The resulting pixel values are used as the patch descriptor. This normalization strategy is selected because it generates similar descriptors regardless of the cell fluorescence level. The resulting models can be found in Fig 3. After image acquisition, the classifier is applied to every possible 9x9 image window. For each patch, the previously described descriptor is extracted in order to find the class with the highest posterior probability given the learned mean and covariance matrix of each class. Once the LDA algorithm is applied to every extracted descriptor, a threshold is applied to the resulting probability map, resulting in a binary map in which each spot represents one or more objects. This threshold is set using cross validation to minimize detection of false positives while having a true positive rate above 99%.

### Heterogeneous EM

Fluorescence intensity and diameter quantification is carried out for each detected spot using a novel variant of the EM algorithm. This new variant fits one gaussian distribution to each detected spot and a uniform distribution to the background within a small patch. Each gaussian distribution enables signal quantification while the uniform distribution models the local background level which, if the patch is small enough, should tend towards uniformity.

Each patch is assumed to be generated by a probability density function representing the likelihood of a pixel being hit by a photon. In this approach we model the probability density function as a mixture model where each cell and the background are represented by a component of the mixture. Under the assumption that micron sized cells in low magnification images have a gaussian appearance, recovering cell fluorescence and diameter is equivalent to fitting a gaussian PDF to the cell and analyzing the fitted parameters. The integral of the likelihood function applied to the image was used as the surrogate for fluorescence intensity and the full width at half maximum of the gaussian function as the surrogate for cell diameter; this fitting was carried out using a modification to the well described EM algorithm [28].

Traditionally, the EM algorithm is performed using a mixture of gaussians. However, such model does not fit the reality of the images which exhibit a uniform random background noise, as shown in Fig 3. We have therefore modified the traditional EM algorithm to include a uniform distribution in addition to the mixture of gaussians, in order to account for image noise. The EM algorithm is performed by calculating the contribution of each distribution to each pixel. Traditionally, this contribution is calculated for each photon reaching a pixel, which would require calculating the contribution for a pixel as many times as the intensity level of the pixel. Instead of following this redundant approach, the contribution is calculated once per pixel and, when the maximization step is performed, the contribution of each pixel is multiplied by its intensity level *I*(*x*).

To model a bright spot and background, the contributing functions are a gaussian PDF (Eq 1) and a uniform PDF (Eq 2), respectively. This way, if the algorithm is fitting *N* gaussians, *f*_*c*_ will be a normal distribution for *c* from 1 to *N*, and *f*_*c*_ will be a uniform distribution for *N* + 1.

$$f_{c}\left(x\right)=\frac{e^{-\frac{1}{2}{\left(x-\mu \right)}^{T}\Sigma^{-1}\left(x-\mu \right)}}{\sqrt{{\left(2\pi \right)}^{2}\left|\Sigma \right|}}\;;\;c\;=\;1‥N$$ (1)

$$f_{c}\left(x\right)=\frac{1}{width_{im}*height_{im}}\;if\;c\;=\;N\;+\;1,$$ (2)

in the equations, *μ* represents the mean of the distribution, Σ denotes the covariance matrix, and *width*_*im*_ and *height*_*im*_ represent the image dimensions. In practice, when processing a field of view, the algorithm fits a single uniform distribution to the whole image and a gaussian distribution to each cell; although the fitting algorithm is not limited to this global optimization approach, this simplifies the recursive split operations. When applying this algorithm, the user should decide whether to apply it globally, assuming a uniform background, or locally so it can handle heterogeneity in the background (e.g., unbound aggregates of fluorescent labels or debris). The expectation step measures the contribution of each component of the mixture to the generation of population elements. In our case, there is only one population element per pixel and the mixture of probability density functions is heterogeneous, having a gaussian distribution for each cell, and one uniform distribution to model image noise. Eq 3 summarizes this process, where *π*_*c*_ represents the mixing factor and *θ*_*i*_ represents the parameters of the probability density function *i*.

$$z_{c}\left(x\right)=\frac{\pi_{c}f_{c}\left(x|\theta_{c}\right)}{\sum_{n}\pi_{n}f_{n}\left(x|\theta_{n}\right)}$$ (3)

The maximization step fits each distribution to maximize its contribution over the data it is modeling. When maximizing the uniform distribution only Eq 4 has to be computed, whereas when maximizing the gaussian distributions Eqs 4, 5, and 6 have to be computed in order to update the gaussian mixture parameters.

$$\pi_{c}^{i+1}=\frac{\sum_{n}z_{c}\left(x_{n}\right)I\left(x_{n}\right)}{\sum_{n}I\left(x_{n}\right)}$$ (4)

$$\mu_{c}=\frac{\sum_{n}z_{c}\left(x_{n}\right)I\left(x_{n}\right)x_{n}}{\sum_{n}z_{c}\left(x_{n}\right)I\left(x_{n}\right)}$$ (5)

$$\Sigma_{c}=\frac{\sum_{n}z_{c}\left(x_{n}\right)I\left(x_{n}\right)\left(x_{n}-\mu_{c}\right){\left(x_{n}-\mu_{c}\right)}^{\prime }}{\sum_{n}z_{c}\left(x_{n}\right)I\left(x_{n}\right)}$$ (6)

After the iterative process has converged, the likelihood over each of the gaussian components is computed, which represents the pixel-wise cell probability. This process creates a probability map that, when multiplied by the image, outputs a new image in which only cells are present. The last remaining step involves summing pixel intensities over each cell domain which gives the final fluorescence estimation. This process is summarized in Eq 7. In order to estimate the diameter a cell, the pixel size was multiplied by the full width at half maximum of the fitted gaussian distribution.

$$Fluorescence_{c}=\sum_{x}z_{c}\left(x\right)I\left(x\right)$$ (7)

### Outlier analysis

Since white blood cells and small cell clusters follow certain shape and size constraints, any event that does not conform to these constraints can reasonably be discarded. The size and shape of the event is measured through the covariance matrix of each fitted gaussian. There are three cases that indicate that a particular event is invalid. First, when the variance along some axis is very small, an extreme case would be when 3*σ* < 1, meaning that the gaussian does not even cover a single pixel. Second, opposite to the first case, when a gaussian PDF covers a section of the image larger than any expected cell or any cell cluster with more than three cells, which occurs when the algorithm is attempting to fit the background instead of a single bright spot. Third, when the EM algorithm fits a correlated set of pixels wherein the resulting covariance is degenerate, which may arise from fitting a small background region using a gaussian distribution. All these cases are rejected from further processing.

Siding window classifiers often detect clusters of few cells as a single event. To ensure all clustered spots are properly analyzed, a post processing step is introduced that takes advantage of our fitting procedure. This step recursively splits the gaussian distributions of clusters in order to fit each individual spot. Contrary to many split and merge implementations, our algorithm is completely deterministic since it only uses image information to calculate split magnitude and orientation. To calculate the direction and magnitude of the splitting, we assume that bright spots have similar shape and brightness. When one gaussian distribution is fitting two bright spots in close proximity, the mean of the distribution lies between both bright spots, the direction of maximum variance is the axis that goes through both spot centers and the variance of the gaussian is a function of the distance between the bright spots. Eigenvector decomposition of the covariance matrix allows for the computation of the split direction and magnitude. The split direction is the one in which the fluorescence has the largest variance, that is the eigenvector with the largest eigenvalue. The split magnitude is calculated such that two separate equal gaussian spots have been fitted by a single gaussian. The split magnitude is the distance between the fitted gaussian mean, and each of the real gaussian spots means; this can be expressed as the standard deviation along the axis of largest variance or, in other words, the square root of the largest eigenvalue. After splitting, the H-EM algorithm is re-initialized, but with two gaussian functions, each with their different covariance matrix, thus enabling the analysis of different sized cells. The splitting method is applied recursively until convergence, measured using the Bayesian Information Criterion (BIC) [29]. For each iteration, the gaussian with the largest eigenvalue is split and the BIC calculated before and after the split are compared. When the difference between the BIC of the current and previous iteration is below a certain threshold, the splitting of the gaussian PDF is halted and the results prior to the latest split operation are kept for further processing.

## Results

### Algorithm performance

To validate the algorithm performance with respect to other microscopy analysis algorithms, and showcase the feasibility of cell astronomy, experiments with both synthetic beads and stabilized leukocytes were conducted.

#### Detection performance

The detection performance on unsorted cell images was quantified by comparing the output of the classifier to the manual annotations of cell locations provided by an expert. To carry out the comparison, cell and background patches were extracted and the algorithm was evaluated using cross validation. The resulting TPR (true positive rate) and FPR (false positive rate) were 0.9780 and 5.1629e-04, respectively.

#### Photometry performance comparison

We compared our fluorescence intensity estimation to that of DAOSTORM in sorted cell samples. DAOSTORM was used as reference since it is based in DAOPHOT [16], which is one of the most cited photometry algorithms applied in astronomy, and is one of the best performing algorithms in a recent evaluation of software packages for single-molecule localization microscopy [2]. We used the correlation coefficient among both methods as the figure of merit, since fluorescence does not need to be estimated in an absolute scale for cytometry analyses. To this end, we prepared three kinds of samples, each containing a pure population of either granulocytes, monocytes, or lymphocytes. Four fields of view were acquired for each sample. When analyzing the samples with DAOSTORM, a background sigma of 17 and a threshold of 4 was used for the spot detection routine.

**Fig 4:**
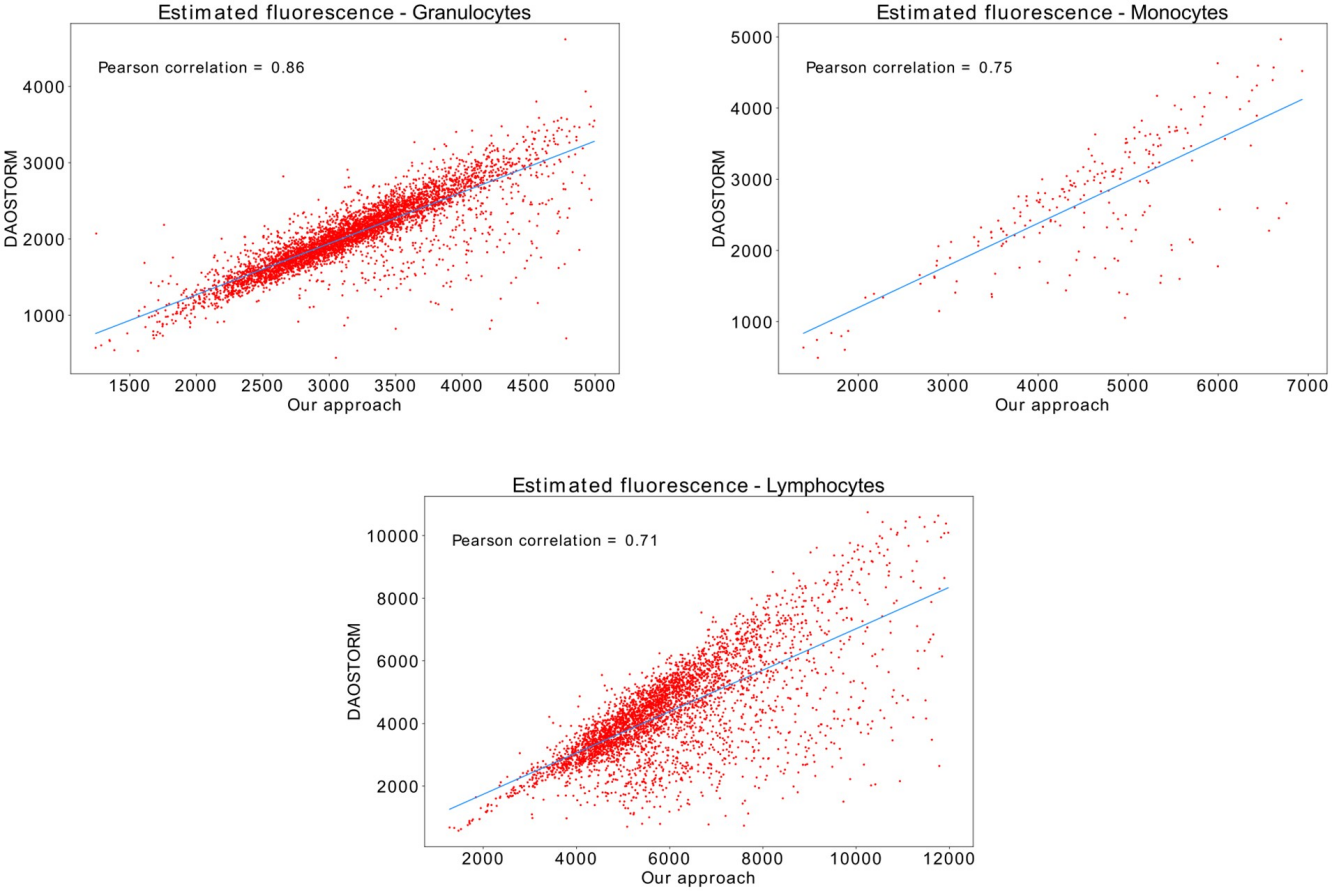
Photometry comparisons. Scatter plots showing the correlation between our approach and DAOSTORM for granulocyte, monocyte, and lymphocyte populations. The blue lines in the plots represent the linear fit of the two measurements.

The scatter plots in Fig 4 show the correlation between our approach and DAOSTORM when estimating the fluorescence intensity of granulocytes, monocytes, and lymphocytes. The correlation coefficients are 0.86, 0.75, and 0.71 respectively. The average correlation across cells is 0.77. Such moderate correlation coefficients can be due to the fact that DAOSTORM is designed to account for unresolved objects, while the cells imaged by our system span several pixels. This explanation is coherent with the fact that the diameter of granulocytes range from 10-15 *μm*, while the diameter of monocytes ranges from 15-30*μm* and lymphocytes range from 7-15 *μm*. The correlation between our method and DAOSTORM is larger when the variability in cell diameter is smaller. Since DAOSTORM uses a single PSF to fit all bright spots, variations in spot size will increase the fitting error, and subsequently increase the error in estimated fluorescence.

To assert which method is generating more reliable results, we manually analyzed two lymphocyte subsets using aperture photometry. The first subset is comprised of the 30 cells for which both methods disagreed the most, and the second subset is comprised of 30 random cells. The first subset is aimed at deciding whether DAOPHOT tends to underestimate the fluorescence of lymphocytes, or our approach tends to overestimate it, while the second subset will provide an overall accuracy estimate when compared to manual analysis. The analysis of the 30 cells with the most disagreement shows that our approach has an error rate of 10.47% while DAOPHOT has 67.27%, which indicates that DAOPHOT is underestimating the fluorescence of a significant number of cells. On the second subset, the analysis of 30 random cells shows that our approach has an overall error rate of 8.8% while DAOPHOT has an error rate of 19.74%.

#### Splitting performance

**Fig 5:**
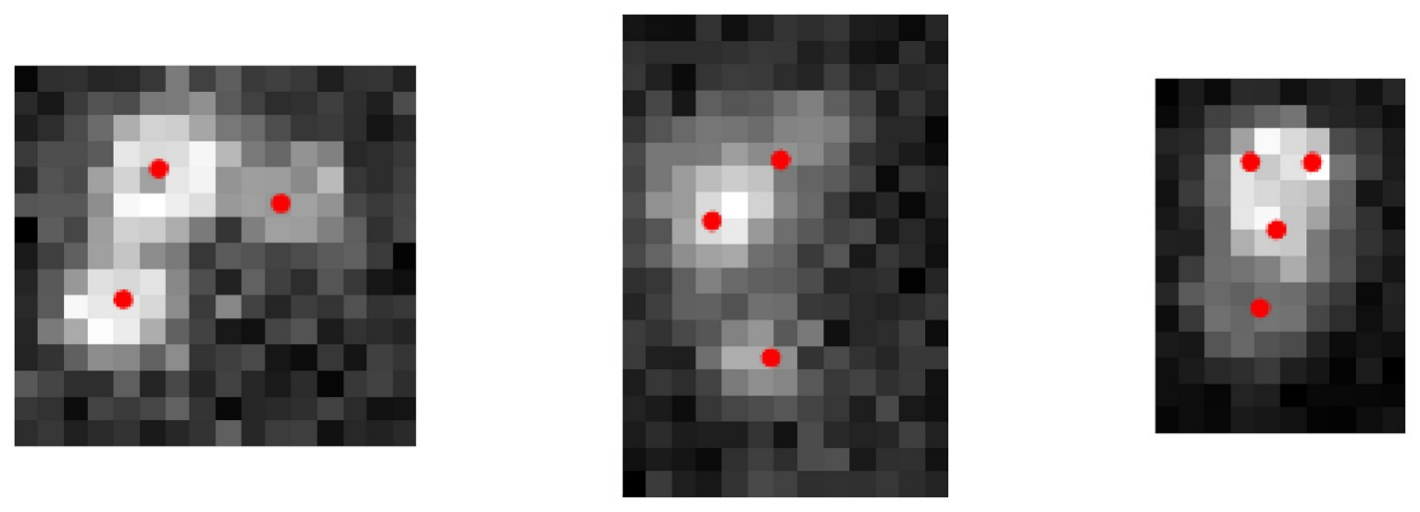
Clusters may be composed of cells having different diameters and fluorescences. In the first two examples the cells were identified correctly, but in the third example the algorithm overfitted 4 cells instead of 2. The mean of the fitted gaussians are represented by red dots.

The splitting performance on unsorted cell images was quantified by comparing manual annotations of clusters of two or three cells to the output of the algorithm. The algorithm was initialized with a single gaussian in the center of the bounding box enclosing the cluster. After this initial setup the recursive splitting algorithm was run until convergence. The results indicated that 80.18% of the clusters were correctly analyzed. Fig 5 illustrates some challenging samples that present nonuniform cell diameters and fluorescence levels.

### Application to cell astronomy

After showing that the proposed image analysis pipeline can detect events, estimate their fluorescence better than standard algorithms, and recursively split clusters, we proceeded to characterize its performance with calibration beads and a well-characterized biological sample.

#### Bead analysis performance

We imaged and analyzed samples containing beads of MESF levels 2, 3, and 4, with the goal of characterizing inter-cluster separation and the influence of signal intensity on diameter estimation. Three samples, one for each bead intensity level, were prepared and imaged individually. The mean diameters of the beads was of 7.52*μm*, with a coefficient of variation of 4.8%, as specified by the manufacturer via Coulter Principle.

Table 1 shows the mean fluorescences, diameters, and image SNRs of the analyzed bead groups. The estimated physical diameter for all the three bead types falls in the expected range specified by the manufacturer, 7.1–7.9*μm*. The average recovered bead diameter is 7.6*μm* with a coefficient of variation of 17.44%. The data shows that there is 5.33% increase in the estimated diameter of MESF level 2 beads with respect to MESF level 4 beads, probably due to the small SNR of those images.

**Table 1:** Mean fluorescence and diameter of different groups of beads. Reference MESF refers to bead intensity as measured by the manufacturer. The coefficients of variation for each measurement are shown in parenthesis.

MESF	Reference MESF	SNR	Mean Fluorescence	Diameter (*μm*)
4	624,803 (0.42%)	102.0	4851.3 (42.5%)	7.5 (16.7%)
3	138,201 (1.24%)	23.5	1197.4 (52.1%)	7.7 (16.0%)
2	18,882 (2.07%)	7.8	372.3 (42.5%)	7.9 (18.6%)

**Fig 6:**
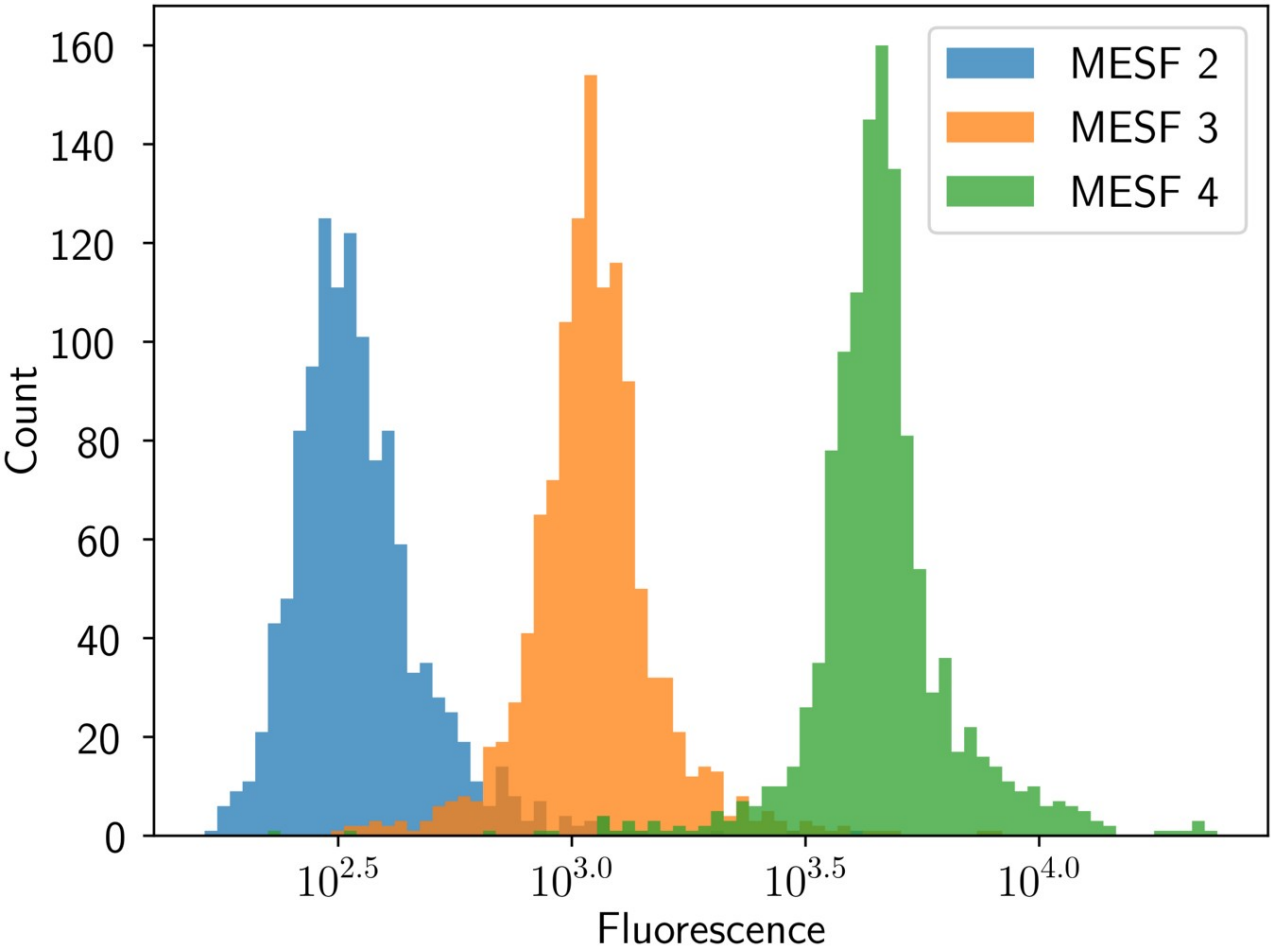
Bead performance. Beads of MESF levels 2, 3, and 4 were analyzed, and the estimated fluorescences were combined into a single histogram. This histogram shows the algorithm is capable of separating the three bead types with little overlap between the histogram of different MESF levels.

Finally, the correlation coefficient between the estimated fluorescence and the reference bead MESF fluorescence is *R*^2^ = 0.9998 (*p* = 0.008), showing good linearity against the reference standard. The coefficients of variation of each bead type are larger than those of the reference standard, being of 49%, 52%, and 42% for MESF beads of levels 2, 3, and 4, respectively. Despite having large CVs, the three bead populations can be distinguished, as shown intuitively in Fig 6 and numerically through the hypothesis testing performed in Table 2.

**Table 2:** Results of t-test and Cohen’s d for the fluorescence intensity of MESF level 2 against MESF level 3, and MESF level 3 against MESF level 4.

Populations	p-value	t-value	Cohen’s d
2-3 MESF	< 0.0001	-61.71	-1.75
3-4 MESF	< 0.0001	-114.80	-2.30

#### Cell population analysis performance

We sorted the biological sample into granulocytes, monocytes, and lymphocytes using flow cytometry. These pure population samples were used to compare the measurements from the cell astronomy system (microscope and analysis pipeline) to the reference standard flow cytometer.

**Fig 7:**
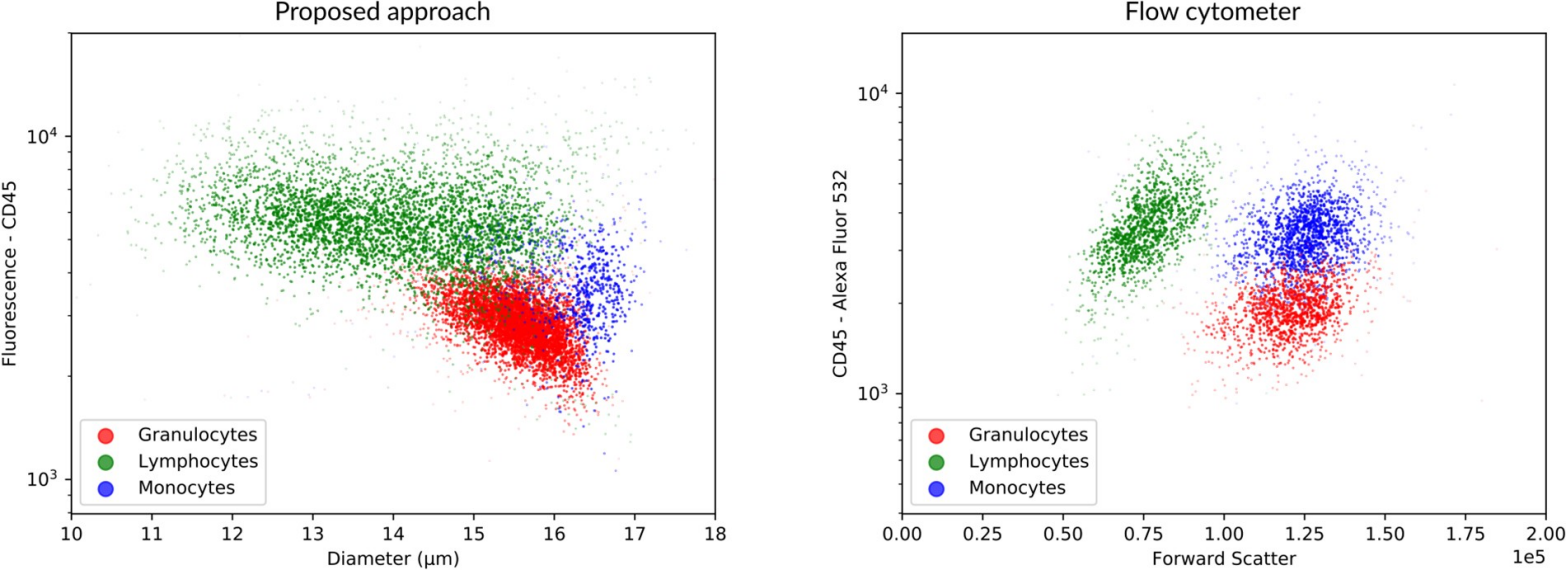
Cell performance. Sorted samples were analyzed using a flow cytometer and our approach. The results of the two methods demonstrates similar relative positions of the 3 cell groups. Monocytes show the largest average diameter while lymphocytes show the largest diameter variation. Granulocytes present the lowest CD45 fluorescence intensity, while lymphocytes present the highest fluorescence intensity and the monocyte fluorescence intensity is intermediate to the other two.

Fig 7 shows the resulting scatter plots obtained from the cell astronomy system and the flow cytometer. This test underlines the importance of the diameter estimation when analyzing cell samples; because the CD45 fluorescence intensity of the monocyte population falls between the intensity of the granulocytes and lymphocytes, it is unfeasible to identify the three groups using fluorescence alone. The scatter plots are not identical due to the different origin of the measurements, which is particularly evident for the diameter measurements. Indeed, flow cytometry measures forward cell scatter, and uses such measurement as a surrogate for diameter, whereas the cell astronomy system measures actual diameter. Despite such differences, three different clusters are observed in both scatter plots, showing the ability to distinguish leukocyte subtypes using both systems.

Measured CD45 fluorescence intensity was compared to previous reports [30], which used a similar flow cytometer (FACSCanto II, Becton Dickinson Bioscience, San Jose, CA, USA). Both studies show that lymphocytes have the highest CD45 fluorescence intensity while granulocytes have the lowest. There is also agreement on the relative position of the three groups; lymphocytes and granulocytes show little overlap, while monocytes fall between these two groups. The diameters estimated by our algorithm were compared to the expected relative cell diameters reported by [31]. Our results agree that monocytes have the highest average diameter, with mean and standard deviation of 16.1 ± 0.67*μm*, lymphocytes have the highest diameter variation 14.0 ± 1.31*μm*, and neutrophils (the most abundant kind of granulocytes) have a diameter smaller than monocytes and similar to the largest half of the lymphocytes, 15.5 ± 0.56*μm*.

## Discussion

Cellular astronomy was proposed to achieve high-throughput imaging cytometry; however, the interpretation of such low-resolution images of fluorescent cells with the goal of estimating their diameter and fluorescent labeling is a challenging task. In this work we have proposed an algorithmic pipeline for image analysis and interpretation, enabling simultaneous accurate photometry and estimation of the cell diameter.

To detect and identify cells in the image we have used a model-based approach that shows a high true positive rate (TPR) of 0.9780 while rejecting irrelevant events, with a false positive rate (FPR) of 5.1629e-04. Such model-based cell detection method is on par with state of the art methods [5]. The high true positive rate ensures that the majority of the cells are located, while having a low false positive rate ensures that only cells or small cell clusters are detected.

We have shown that the proposed pipeline can estimate fluorescence with lower error, with respect to manual photometry, than DAOSTORM. Indeed, the results suggests that DAOSTORM has the tendency to underestimate the fluorescence of cells. Additional tests using microbeads show that the proposed approach can estimate the fluorescence intensity of MESF beads of levels 2, 3, and 4 with high linearity and achieve inter-cluster separation.

Further, the system is able to recover the diameter of the objects under inspection. The average recovered bead size is of 7.6*μm* while the reference standard is of 7.5*μm*. The coefficient of variation of bead diameters estimated with our system is larger than that of the specifications of the beads used regularly for flow calibration. Large coefficients of variation can be explained by the low-resolution images we acquire. The diameter of a 7.5*μm* object is mapped to a low number of pixels (3-4). An error of one pixel in diameter estimation incurs in an error between 25% and 33%. Due to partial volume effects, such error is to be expected. It is also possible to recover the diameter of cells, which, in our system are 14.0 ± 1.3*μm* for lymphocytes, 15.5 ± 0.56*μm* for granulocytes and 16.12 ± 0.67*μm* for monocytes. Standard diameters for such cells are 7–12*μm* for lymphocytes, 10–20*μm* for granulocytes and 10–18*μm* for monocytes [32]. Granulocytes and monocytes are within the expected range. Lymphocytes appear larger in our system than the size that is cited in the literature. Such effect could be due to the particularities of the sample, the sample preparation method, or the H-EM algorithm. Discerning between such explanations require reference standard size measurements that are not currently available.

We have described a method to refine detections for clustered cells in contrast to the common approach in flow cytometry which excludes doublets from the final analysis [1]. The likelihood of encountering doublets or triplets using cellular astronomy may also be tuned by carefully selecting a sample preparation methodology. However, there is a tradeoff between the probability of doublets appearing and the density to which the sample can be prepared, which has implications on instrument throughput.

Using size and fluorescence estimates, we compared the results of using our approach to those of using a flow cytometer. Fig 7 shows the 3 differentiated cell groups for both our system and the flow cytometer. As flow cytometry does not measure the same physical properties as our algorithm, there is an understandable difference between the two approaches. However, the three groups are clearly differentiated in both approaches.

## Conclusion

The estimation of cell fluorescence and size from cellular astronomy images is challenging due to the inherent low-resolution properties of the images, different cell sizes, and the appearance of small cell clusters. We have developed H-EM, an algorithm that estimates cell size and fluorescence, and is robust to such image properties. We have performed extensive evaluation of the proposed method using MESF beads and fluorescent stained leukocytes, showing that H-EM’s intensity estimation is more accurate to manual measurements than that of established algorithms, such as DAOSTORM. Further, H-EM recovers cell diameter estimations, enabling cell cluster separation.

Our future work will focus on the development of fitting methods powerful enough to handle more challenging scenarios such as dark field images, where the background is noisier because of the light scattered by the debris. An algorithm that presents a better way of handling the background would not only increase the brightness and diameter estimation performance for fluorescence imaging, but also enable its application to a wider set of problems. Another important improvement would be to use a more configurable shape prior, which would improve the performance of the algorithm when analyzing non-gaussian shapes which may appear both in cells and in standard astronomy.
